# Health insurance coverage and maternal healthcare services utilization in Jordan: evidence from the 2017–18 Jordan demographic and health survey

**DOI:** 10.1186/s13690-021-00605-4

**Published:** 2021-05-19

**Authors:** Petula Fernandes, Emmanuel Kolawole Odusina, Bright Opoku Ahinkorah, Komlan Kota, Sanni Yaya

**Affiliations:** 1 Interdisciplinary School of Health Sciences, University of Ottawa, Ottawa, Ontario Canada; 2 Department of Demography and Social Statistics, Federal University, Oye, Ekiti Nigeria; 3 The Australian Centre for Public and Population Health Research (ACPPHR), Faculty of Health, University of Technology Sydney, Ultimo, Australia; 4 School of International Development and Global Studies, Faculty of Social Sciences, University of Ottawa, 120 University Private, Ottawa, ON K1N 6N5 Canada; 5 The George Institute for Global Health, The Imperial College London, London, UK

**Keywords:** Maternal health care, Health insurance coverage, Jordan, Demographic and health surveys

## Abstract

**Background:**

Despite the relationship between health insurance coverage and maternal healthcare services utilization, previous studies in Jordan on the use of maternal healthcare services have mainly focused on patterns and determinants of maternal healthcare services utilization in Jordan. Therefore, this study investigated the relationship between health insurance coverage and maternal healthcare services utilization in Jordan.

**Methods:**

This study used secondary data published in 2017-18 Jordan Demographic and Health Survey on 4656 women of reproductive age (15–49 years). The independent variable was health insurance coverage and the outcome variable was maternal healthcare services utilization, measured through timing of first antenatal visit, four or more antenatal care visits, and skilled birth attendance. The data were analyzed using descriptive statistics and binary logistic regression.

**Results:**

Out of the total number of women who participated in the study, 38.2% were not covered by health insurance. With maternal healthcare utilization, 12.5%, 23.2%, and 10.1% respectively, failed to make early first antenatal care visit, complete four or more antenatal care visits and have their delivery attended by a skilled worker. After controlling for the socio-demographic factors, health insurance coverage was associated with increased odds of early timing of first antenatal care visits and completion of four or more antenatal care visits (aOR = 1.33, *p* < 0.05, aOR = 1.25, *p* < 0.01, respectively). However, women who were covered by health insurance were less likely to use skilled birth attendance during delivery (aOR = 0.72 *p* < 0.001).

**Conclusions:**

Jordanian women with health insurance coverage were more likely to have early first antenatal care visits and complete four or more antenatal care visits. However, they were less likely to have their delivery attended by a skilled professional. This study provides evidence that health insurance coverage has contributed to increased maternal healthcare services utilization, only in terms of number and timing of antenatal care visits in Jordan. It is recommended that policy makers in Jordan should strengthen the coverage of health insurance in the country, especially among women of reproductive age in order to enhance the use of maternal healthcare services in the country.

## Background

Sustainable Development Goal 3 (SDG-3) focuses on ensuring healthy lives and promoting well-being for all, at all ages [1]. Target 3.8 of SDG-3 is centered on achieving universal health coverage (UHC) through financial risk protection and access to quality essential healthcare services [1, 2]. This target has two indicators - 3.8.1 on coverage of essential health services and 3.8.2 on the proportion of a country’s population with catastrophic spending on health [3]. It is imperative that both of these indicators are measured together to obtain a clear picture of which demographic groups in a population are unable to access healthcare, those that exhibit underutilization of healthcare services and those that encounter financial hardship due to healthcare expenditures [3]. It is key to stress that all segments of a population should be considered to identify the most disadvantaged communities that experience limited or no healthcare services due to financial constraints. In line with this, is the SDG’s pledge to “leave no one behind” to ensure that the fundamental human right to healthcare is extended to every individual regardless of age, sex, religion, socio-economic status, and life circumstance [4].

The 2017 UHC Monitoring Report revealed that at least half of the world’s population still lacks access to essential health services due to financial constraints [3]. Furthermore, some 800 million people spend more than 10% of their household budget on health and 100 million people are pushed to extreme poverty annually as a result of out-of-pocket health expenses [3]. In Jordan, health insurance providers include the Ministry of Health (MoH), the Royal Military, the University Hospital, the United Nations Refugee Welfare Association (UNRWA), the United Nations High Commissioner for Refugees (UNHCR), nongovernmental insurance, and private insurance [5]. An individual may have more than one form of coverage from the above listed sources. In total, 58% of women and 50% of men aged 15–49 have some type of health insurance coverage, while 42% of women and 50% of men do not have any health insurance [6]. This is still a substantial amount of the population that is left uninsured. Moreover, the Middle Eastern nation has experienced a drastic demographic change within the last decade with the large influx of Syrian refugees from neighboring Syria that have sought asylum in the country since the wake of the Syrian civil war [7]. Further to mention, Jordan also houses more than 2 million Palestinian refugees that have been residing in the country since 1948 [8]. This makes Jordan the largest refugee-hosting country worldwide [9]. Its non-national residents have placed a tremendous burden on the country’s healthcare system and largely remain unaccounted for in the government’s health insurance coverage scheme [10]. The current Jordanian government has established a goal to reach UHC, reduce health inequality and inequity and improve the quality of healthcare services for all its residents [5].

Maternal healthcare (MHC) is an example of an essential healthcare service that needs improvement in the country. MHC services during these periods are essential to the survival and well-being of the mother and infant [11]. The services that are considered standard include: timing of first antenatal care visit (ANC), four or more ANC visits, and skilled birth attendance, Bacillus Calmette-Guerin vaccination, the third dose of diphtheria-tetanus-pertussis vaccine and the measles vaccine [12]. MHC serves as a significant intervention in decreasing maternal morbidity and mortality [13]. Additionally, the utilization of maternal services can be regarded as a prominent indicator of a population’s accessibility to receiving the treatment [14]. While individual-level factors such as socioeconomic situation, educational level and female decision-making power have been studied to assess under-utilization of MHC services, the effect of health insurance coverage has not received adequate attention for women in Jordan, especially within the last 5 years to reflect Jordan’s drastic demographic change [15–17].

Despite the relationship between health insurance coverage and MHC services utilization [18–21], previous studies in Jordan on the use of MHC services have mainly focused on patterns and determinants of MHC services utilization in Jordan [16, 22]. None of these studies looked at the link between health insurance coverage and MHC services utilization, while controlling for the effect of other important socio-demographic characteristics. Apart from this, the previous studies on MHC were conducted more than two decades ago and none of them used a nationally representative data such as the Demographic and Health Survey data. To fill the gap in literature, this study aims to examine the association between health insurance coverage and the utilization of MHC services among women in Jordan. This, in turn, will inform the design and implementation of evidence-based and population specific interventions and social policies that will work towards achieving UHC to facilitate access to quality MHC services to all women in need in Jordan. The findings will provide guidance for policy-makers in Jordan to address the substantial disparity in MHU among the twelve governorates by creating a comprehensive and equitable national health insurance scheme. Results of this secondary analysis will serve to inform the dire need to achieve universal health coverage as addressed in the 2017 Universal Health Coverage Monitoring Report [3] in order to meet one of the targets outlined in SDG-3 by the UN [1, 2]. In addition, this study’s findings also provide guidance for policy-makers in Jordan to address the substantial disparity in MHU among the twelve governorates by creating a comprehensive and equitable national health insurance scheme.

## Conceptual framework

This study adopts the Health Care Services Utilization Model proposed by Anderson in the 1960s to examine the role health insurance coverage and other socio-demographic characteristics of women play in MHC services utilization in Jordan. The framework provides an explanation on the conditions that either promote or hinder the utilization of health care services [23]. This model identified three main conditions under which an individual will use or not use a health care service. These factors are the predisposing factors; enabling factors and need for care factors. Predisposing factors describe the demographic, social structure, and health belief characteristics of an individual. The demographic characteristics may include the sex of the individual, the age, and the marital status of the individual. Social Structure consists of education, ethnicity, and occupation while health belief factors are the values, attitudes of health care service providers, and knowledge about health. Enabling factors are the resources or means that are available to an individual to seek health care services. The enabling factors include income and health insurance coverage. Need for care factors refer to how an individual’s perceive their own general health and functional condition, as well as their familiarity with the signs and symptoms of ill health, agony and concerns about their health [24].

## Methods

### Data source

Data for this study were collected from the Jordan Demographic and Health Survey (DHS) conducted in 2017–18. The study included a sample of 4656 childbearing women aged (15–49 years). The sample was derived from childbearing women who had complete cases on all the variables of interest in this study. In this study, we treated missing values by using complete cases for our analysis. Hence, the unit of analysis in the study was childbearing women. The survey captures data on various socio-demographic characteristics of participants’ and health insurance coverage. The survey also captures data on the participants’ use of key maternal health services distributed among the factors of socio-demographics characteristics and health insurance coverage. The 2017–18 Jordan DHS survey is a representative household survey consisting of 19,400 residential households and the selected samples reflect the populations from which they were drawn. The survey used a stratified sample, selected in two stages from the 2015 census frame. Stratification was achieved by separating each governorate into urban and rural areas. Data for this study can be accessed via: https://dhsprogram.com/methodology/survey/survey-display-500.cfm.

### Variables and measurements

#### Outcome variable

The outcome variable of interest was MHC services utilization. Variables were selected based on previous literature published on MHC services utilization. As determined by the WHO guidelines on essential MHC services, the following factors were used to measure MHC services utilization: timing of the first antenatal care visit (ANC), completing the recommended number of ANC visits, and giving birth at a health facility under skilled care. First trimester of a pregnancy is the recommended time for pregnant women to have their ANC consultation. Four spaced visits are the recommended number of ANC visits. Lastly, a skilled birth attendance involves having the expectant mother’s delivery be attended by a qualified health worker at a health facility.

#### Independent variables

The independent variable in this study was HIC. This was derived from the question ‘Are you covered by any health insurance?’ The responses were ‘Yes’ and ‘No’. For the purpose of the analysis, the responses were coded as 1 = “Yes” and 0 = “No”. The choice of health insurance coverage as the key independent variable was based on its association with MHC services utilization in previous studies [18–21].

#### Covariates

To adjust the study’s analysis for potential confounding variables, socio-demographic variables such as age groups (15–19, 20–24, 25–29, 30–34, 35–39, 40–44, 45–49), education (no formal education, primary, secondary, post-secondary), occupation (not working, working), marital status (married, widowed/divorced/separated), area of residence (urban, rural), and wealth index (poorest, poorer, middle, richer, richest) were included. In DHS, wealth index is computed using Principal Component Analysis (PCA) based on the household assets and characteristics of the household [25]. Number of births in the past 5 years before the survey was conducted was dichotomized into “one birth” or “2 or more births”. Jordan’s regions were divided according to the country’s 12 governorates: Amman, Balga, Zarqua, Madaba, Irbid, Mafraq, Jerash, Aljoun, Karak, Tafilh, Maan, and Aquaba. These variables were not determined a priori; but were selected based on their availability in the 2017–18 Jordan Demographic and Health Survey, their theoretical relevance and practical significance in previous studies conducted in the country [16, 22].

### Data analysis

The data were analyzed using both descriptive and regression methods with Stata version 14. First, descriptive statistics on the distribution of participants’ socio-demographic characteristics by health insurance coverage and MHC services utilization were performed. For MHC services utilization, timing of first ANC visit, completing the recommended number of ANC visits and giving birth under skilled labour attendance were analyzed. For all the analyses, the level of significance was set at *p* < 0.005 (2-tailed). To check for a high correlation among the independent variables, a test for multicollinearity was carried out using the variance inflation factor (VIF), and the results showed no evidence of high collinearity (Mean VIF = 1.49, Maximum VIF = 2.46, and Minimum VIF = 1.02). Next, a multivariable logistic regression was conducted to examine the independent association between health insurance coverage and the three factors used to measure MHC services utilization. The selection of variables into the regression model was based on the conceptual framework that guided the study and not their statistical significance in the chi-square test. Hence, all variables were included irrespective of their statistical significance in the chi-square test. The generated adjusted odds ratio (aOR) were estimated to assess the strength of the associations and used the 95% confidence intervals (CIs) for significance testing. The analysis catered for the robustness of the standard errors. All frequency distributions were weighted while the survey command (SVY) in Stata was used to adjust for the complex sampling structure of the data in the regression analyses.

## Results

### Prevalence of health insurance coverage and maternal healthcare services utilization

The study sample consisted of 4656 participants. Of all the participants, 1780 (38.2%) reported no health insurance while 2875 (61.8%) were covered by a health insurance scheme of some sort. With MHU, 4076 (87.6%) had early first ANC visits while 580 (12.5%) had late first ANC visits; 3578 (76.9%) had the recommended number of ANC visits while 1078 (23.2%) did not have the recommended number of ANC visits; 4186 (89.9%) were assisted by a skilled attendant during delivery while 469 (10.1%) did not have skilled birth attendance (Table 1).

**Table 1:** Distribution of participants by factors affecting use of maternal health services during pregnancy and birth among women of reproductive age in Jordan using the 2017–18 Jordan DHS (*N* = 4656)

Variables	Timing of the first ANC visit					Completed recommended number of ANC visits					Birth attended by a skilled worker				
	No		Yes		*P*-value	No		Yes		*P*-value	No		Yes		*P*-value
	N	%	N	%		N	%	N	%		N	%	N	%	
**Health Insurance coverage**															
No	284	15.9	1496	84.1	***	449	25.3	1330	74.8		127	7.1	1653	92.9	***
Yes	296	10.3	2580	89.7		628	21.9	2248	78.2		343	11.9	2533	88.1	
**Education**															
No education	12	18.8	52	81.2	***	26	40.8	38	59.3	***	9	13.4	56	86.6	***
Primary	45	14.7	264	85.3		121	39.2	188	60.8		37	11.9	272	88.1	
Secondary	355	14.5	2089	85.5		629	25.8	1814	74.3		284	11.6	2160	88.4	
Post-secondary	168	9.1	1671	90.9		301	16.4	1538	83.6		140	7.6	1699	92.4	
**Age groups**															
15–19	13	8.8	139	91.2	*	41	27.1	111	72.9		27	17.8	126	82.2	
20–24	112	13.5	718	86.5		200	24.1	629	75.9		88	10.6	742	89.4	
25–29	129	9.6	1211	90.4		319	23.8	1020	76.2		125	9.3	1215	90.7	
30–34	158	13.4	1018	86.6		246	20.9	930	79.1		116	9.9	1060	90.1	
35–39	117	14.4	700	85.6		185	22.6	633	77.4		87	10.6	731	89.4	
40–44	49	16.0	257	84.0		78	25.6	227	74.4		25	8.0	281	92.0	
45–49	2	4.8	32	95.2		8	22.7	26	77.3		1	4.3	32	95.7	
**Occupation**															
Not working	525	12.8	3572	87.2		986	24.1	3112	75.9	**	434	10.6	3664	89.4	**
Working	54	9.8	504	90.2		92	16.4	466	83.6		36	6.4	523	93.6	
**Marital status**															
Married	578	12.5	4036	87.5		1061	23.0	3554	77.0	**	466	10.1	4149	89.9	***
Single	2	5.1	39	94.9		17	41.1	24	58.9		4	9.3	38	90.7	
**Area of Residence**															
Urban	538	13.1	3573	86.9		923	22.5	3188	77.6	0.120	371	9.0	3740	91.0	***
Rural	42	7.6	503	92.4		155	28.5	390	71.6		98	18.1	446	81.9	
**Wealth Index**															
Poorest	146	12.1	1065	87.9		408	33.7	803	66.3	***	204	16.8	1007	83.2	***
Poorer	152	13.8	945	86.2		278	25.3	818	74.7		131	12.0	965	88.0	
Middle	133	12.5	929	87.5		199	18.8	863	81.2		89	8.4	972	91.6	
Richer	93	11.4	726	88.6		140	17.1	679	82.9		35	4.2	785	95.8	
Richest	56	12.0	412	88.0		53	11.3	415	88.7		10	2.2	4186	89.9	
**Number of Births in the last 5 years**															
One birth	276	11.2	2198	88.8	***	525	21.2	1949	78.8	***	199	8.0	2275	92.0	***
Two or more	304	13.9	1878	86.1		553	25.4	1629	74.7		270	12.4	1912	87.6	
**Region**															
Amman	304	18.4	1345	81.6	***	262	15.9	1387	84.1	***	68	4.1	1581	95.9	***
Balga	25	10.9	204	89.1		52	22.8	177	77.2		5	2.3	224	97.7	
Zarqua	70	11.3	551	88.7		148	23.9	472	76.1		46	7.4	575	92.6	
Madaba	29	23.5	94	76.5		24	19.4	99	80.6		24	19.6	99	80.4	
Irbid	66	7.0	871	93.0		232	24.8	704	75.2		124	13.2	813	86.8	
Mafraq	34	9.3	335	90.8		138	37.5	231	62.5		92	24.8	277	75.2	
Jerash	15	8.7	152	91.3		48	29.0	118	71.0		34	20.3	133	79.7	
Aljoun	4	3.6	119	96.4		29	23.7	94	76.3		25	20.0	99	80.0	
Karak	11	7.2	147	92.8		47	29.8	111	70.2		21	13.2	137	86.9	
Tafilh	6	7.6	73	92.4		21	26.2	58	73.8		5	6.2	74	93.8	
Ma’an	10	12.0	73	88.0		32	38.1	51	61.9		12	14.8	71	85.2	
Aquaba	6	4.8	113	95.2		44	36.7	75	63.3		15	12.4	104	87.6	
National	580	12.5	4076	87.6		1078	23.2	3578	76.9		469	10.1	4186	89.9	

Here *, ** and *** indicate *p* < 0.05, *p* < 0.01 and *p* < 0.001 respectively

### Distribution of maternal health utilization across health insurance coverage and other socio-demographic characteristics of respondents

Table 1 shows the characteristics of the 4656 participants stratified by utilization of MHC services as per the guidelines set forth by the WHO.

In this study, a higher proportion of those with health insurance coverage had early timing of first ANC visit compared to their counterparts (89.7% vs 84.1%, *p* < 0.001). Timing of the first ANC visit differed across the various socio-demographic characteristics. For example, it was higher among women with a post-secondary education than those with no formal education (90.9% vs. 81.2%, *p* < 0.001). Additionally, it was higher among women in the 45–49 age group (95.2%), working women (90.2%), and women residing in rural areas (92.4%). Additionally, timing of the first ANC visit was higher among the richer and richest on the wealth index, 88.6 and 88.0% respectively, compared to the poorer and poorest, 86.2 and 87.9% respectively. There were proportionally significant variations across the 12 governorates of Jordan, with the Aquaba region showing the highest proportion of women having the proper timing of their ANC visit (95.2%) compared to only 76.5% in the Madaba region (*p* < 0.001).

There was no difference between those with or without health insurance coverage in completing the recommended four ANC visits (74.8% vs. 78.2%, *p* = 0.072). Additionally, age was not a significant factor in seeing a higher or lower completion rate (*p* = 0.401). Women with no formal education were less likely to complete the recommended number of ANC visits (*p* < 0.001). This observation was also true for women making up the poorest quintile on the wealth index (*p* < 0.001). Married women reported higher ANC visits than their single counterparts (77.0% vs. 58.9%, *p* < 0.01). Disparity of completion rate was seen between certain governorates such as 61.9% vs. 84.1% of Maan and Amman respectively (*p* < 0.001).

Lastly, majority of the study population (90%) reported to have delivered at health facilities under skilled attendants. Counter intuitively, a higher proportion of those with health insurance coverage choose not to have a skilled birth, compared to women without health insurance coverage (11.9% vs. 7.1%, *p* < 0.001). Those with no formal education had lower skilled birth rates than their post-secondary level counterparts (86.6% vs 92.4, *p* < 0.001). Age was not a significant factor in determining delivery assistance (*p* = 0.216). Contrary to the results of ANC completion rates, 90.7% of single women had a skilled birth than married women (*p* < 0.001). Women living in rural areas reported lower skilled birth deliveries compared to their urban counterparts (81.9 vs 91.0%, *p* < 0.001). Women of the richer quintile category had their delivery attended by skilled workers in higher proportion compared to those of the poorest wealth quintile (95.8% vs. 83.2%, *p* < 0.001). Occupational status, parity and the region also demonstrated significant differences with skilled birth attendance in this study (*p* < 0.01, *p* < 0.001 and *p* < 0.001 respectively).

### Association between health insurance and maternal healthcare services utilization

Table 2 shows the results of the multivariable logistic regression analysis of the association between health insurance coverage and MHC services utilization. After controlling for the socio-demographic factors, health insurance coverage was associated with increased odds of timing of first ANC visits and recommended completion rate of ANC visits (aOR = 1.33, *p* < 0.05, aOR = 1.25, *p* < 0.01, respectively). However, women who were covered by health insurance were less likely to use skilled birth attendance during delivery (aOR = 0.72 *p* < 0.001).

**Table 2:** Adjusted Odds Ratios (AOR) for the association between health insurance coverage and maternal health service utilization among women of reproductive age in Jordan using the 2017–18 Jordan DHS (N = 4656)

Variables	Timing of the first ANC visit			Completed recommended number of ANC visits			Birth attended by a skilled worker		
	aOR	95% CI	*P*-value	aOR	95% CI	*P*-value	aOR	95% CI	*P*-value
**Health Insurance coverage**									
No	1.0			1.0			1.0		
Yes	1.326	1.069–1.643	*	1.249	1.072–1.455	**	0.718	0.582–0.886	**
**Education**									
No formal education	1.0			1.0			1.0		
Primary	1.500	0.804–2.799		1.452	0.935–2.254		0.674	0.362–1.253	
Secondary	1.290	0.747–2.229		1.764	1.186–2.621	**	0.681	0.383–1.209	
Post-secondary	1.721	0.963–3.075		2.455	1.620–3.722	***	0.744	0.0.410–1.351	
**Age groups**									
15–19	1.0			1.0			1.0		
20–24	0.885	0.482–1.624		1.135	0.796–1.620		0.940	0.611–1.448	
25–29	0.820	0.451–1.490		0.962	0.679–1.365		0.989	0.645–1.515	
30–34	0.763	0.419–1.391		1.120	0.786–1.594		0.972	0.632–1.494	
35–39	0.613	0.334–1.122		1.002	0.699–1.437		0.968	0.623–1.504	
40–44	0.475	0.249–0.905	*	0.972	0.648–1.458		1.330	0.784–2.257	
45–49	0.356	0.131–0.970	*	1.146	0.535–2.454		1.552	0.504–4.784	
**Occupation**									
Not working	1.0			1.0			1.0		
Working	0.976	0.703–1.354		0.978	0.783–1.222		1.228	0.906–1.665	
**Marital status**									
Married	1.0			1.0			1.0		
Single	0.700	0.283–1.733		0.385	0.205–0.726	**	0.757	0.323–1.775	
**Area of Residence**							1.0		
Urban	1.0			1.0			1.0		
Rural	1.149	0.887–1.488		0.993	0.843–1.171		0.778	0.638–0.947	*
**Wealth Index**									
Poorest	1.0			1.0			1.0		
Poorer	1.141	0.885–1.471		1.201	1.015–1.420	*	1.428	1.159–1.760	**
Middle	1.067	0.808–1.409		1.539	1.270–1865	***	1.671	1.312–2.130	***
Richer	1.223	0.864–1.731		1.555	1.221–1.980	***	2.284	1.608–3.242	***
Richest	1.680	1.003–2.815	*	1.641	1.127–2. 391	*	6.572	2.634–16.401	***
**Number of Births in the last 5 years**									
One birth	1.0			1.0			1.0		
Two or more	0.678	0.558–0.824	***	0.752	0.660–0.856	***	0.803	0.681–0.947	**
**Region**									
Amman	1.0			1.0			1.0		
Balga	1.991	1.274–3.112	**	0.781	0.541–1.127		2.379	0.993–5.700	
Zarqua	2.472	1.669–3.663	***	0.921	0.667–1.272		0.504	0.297–0.855	*
Madaba	0.703	0.496–0.996		0.946	0.663–1.349		0.279	0.166–0.470	***
Irbid	2.980	1.931–5.598	***	0.639	0.464–0. 881	**	0.372	0.222–0.625	***
Mafraq	2.653	1.789–3. 934	***	0.469	0.346–0.635	***	0.230	0.141–0.376	***
Jerash	2.266	1.496–3.431	***	0.547	0.396–0.755	***	0.276	0.166–0.457	***
Aljoun	6.144	3.433–10.995	***	0.643	0.460–0.898	*	0.248	0.149–0.413	***
Karak	2.447	1.464–4.090	**	0.445	0.312–0.636	***	0.429	0.243–0.757	**
Tafilh	2.312	1.490–3.588	***	0.576	0.413–0.805	**	0.997	0.547–1.817	
Ma’an	1.932	1.226–3.045	**	0.340	0.242–0.479	***	0.361	0.209–0.623	***
Aquaba	3.879	2.271–6.627	***	0.317	0.228–0.440	***	0.365	0.211–0.631	***

Here *, ** and *** indicate *p* < 0.05, *p* < 0.01 and *p* < 0.001 respectively

Early timing of first ANC visit was lower among older women (aOR = 0.36, *p* < 0.05) and women with two or more births (aOR = 0.68, *p* < 0.001) compared to younger women and women with one birth. Higher odds of early first ANC visit was observed among richest women (aOR = 1.68, *p* < 0.001) and women in all other regions of the country compared to poorest women and women who lived in the Amman region, respectively.

Having a post-secondary education (aOR = 2.46, *p* < 0.001) and richest wealth index (aOR = 1.64, *p* < 0.05) increased the likelihood of completing the recommended number of ANC visits significant association with completing the recommended number of ANC visits (aOR = 2.46, *p* < 0.001). However, lower odds of completing the recommended number of ANC visits was found among single women compared to married women (aOR = 0.39, *p* < 0.01); women with two or more births compared to one birth (aOR = 0.75, *p* < 0.001) and in all other regions compared to Amman.

The odds of skilled birth attendance increased with wealth index, with richest women having the highest odds compared to poorest women (aOR = 6.57, *p* < 0.001). The odds of skilled birth attendance was lower among women who lived in the rural areas (aOR = 0.78, *p* < 0.001), those with two or more births (aOR = 0.80, *p* < 0.01) and those who lived in all other regions of the country compared to those who lived in urban areas, those with one birth and those who lived in the Amman region, respectively.

## Discussion

This study reports the prevalence of health insurance ownership among Jordanian women across various socio-demographic backgrounds in addition to the association between health insurance coverage and the three key maternal health services; timing of first ANC visit, meeting the recommended four ANC visits and having a skilled birth attendance. This is the first comprehensive study to discuss the association between health insurance ownership and maternal health utilization in Jordan using data from 2017 to 18.

Overall, 38.2% of the study participants reported having no health insurance coverage in the present study. Findings on the prevalence of health insurance coverage is similar but slightly higher than the prevalence obtained in a recent study, where the coverage of health insurance was about 27% [26]. The possible reason for the differences in findings could be the study population and sample size. With MHC services utilization, 12.5, 23.2, and 10.1% respectively, failed to make early first antenatal care visit, complete the recommended number of antenatal care visits and have their delivery attended by a skilled worker. Findings on the prevalence of MHC services utilization is in line with previous studies [27, 28]. The high prevalence of MHU in the country could be attributed to the improvement in maternal healthcare through universal health coverage and the implementation of health insurance in the country [5, 6] and general improvement in access to MHC services utilization through the provision of health facilities and presence of health professionals in those health facilities [29].

After controlling for the socio-demographic factors, we found that health insurance coverage was associated with increased odds of timing of first ANC visits and recommended completion rate of ANC visits. Similar findings were obtained in previous studies [18–21]. The possible reason for the finding could be that health insurance coverage reduces the rates of out-of-pocket payments associated with the use of MHC services utilization. Such payments may pose as financial barriers to women when accessing maternal healthcare services. Interestingly, women who were covered by health insurance were less likely to use skilled birth attendance during delivery. This finding is contradicts the findings of previous studies that found a positive association between health insurance coverage and skilled birth attendance [30, 31]. Further studies are needed to unearth the possible reasons for the negative association between health insurance coverage and skilled birth attendance during delivery in Jordan.

Age, wealth index, region, and parity were all positively associated with timely ANC initiation. This result ties well with a study that examined determinants of ANC attendance among Jordanian women living in disadvantaged communities [32]. Being insured gives women a valid reason to make use of available ANC services. As hypothesized, being insured does yield uptake of maternal services as women are not left financially strained in doing so. Possessing higher education informs women on the necessity of initiating an ANC visit within the first trimester. Younger women and women with higher parity also adhered to the recommended timing of ANC initiation. There was regional variation in this study among Jordan’s twelve governotes. Aljoun, Aquaba and Irbid all fared high in proper ANC initiation while Madaba scored the lowest. This study recommends looking at the strategies that have been effective in promoting a timely ANC visit in the former three governorates. Occupation, marital status, area of residence and wealth index had no influence on the timely starting of ANC visits in this study. Considering that ANC is provided free of charge in Jordan, this may explain the non-significant associations [32].

This analysis found evidence for education, marital status, wealth index and parity influencing respondents to completing the four recommended ANC visits. As observed with the first key maternal health service, educated women are more informed of pregnancy-related risks and will thus adhere to completing the required amount of ANC visits in comparison to lower and non-educated women. In line with other studies [5, 16, 17], education proves to be a strong determinant in uptake of maternal health services. Being employed was a positive indicator for women fulfilling advised ANC visits as having a stable job grants financial security, thus allowing expectant mothers to be able to afford maternal services. Married women were more likely to meet the four standard visits than their single counterparts. This can be attributed to married women receiving financial and emotional support from their spouses to partake in maternal services. Women placed on the richer and richest category on the wealth index met the ANC visit completion rate. Above average financial standing readily equips women to be able to afford these services. Lastly, parity was positively associated with ANC visits; this can be owed to women having more experience and acquired knowledge from prior pregnancies. Once again, regional variation was observed among the dozen governorates. The Ma’an region saw the lowest completion rate, in which less than three-quarters of participants failed to meet the recommended ANC visits. Associations with health insurance coverage, age and area of residence did not reach statistically significant levels in the present analysis. As with the preceding section, this may be attributable to Jordan offering ANC services at no charge.

The findings in this study revealed that area of residence, wealth index and parity influenced the study participants to have a skilled birth attendance. Educated expectant mothers are likely to be aware of birthing-risks and may opt to have their delivery under the care of a skilled worker [17–21, 33]. As discussed in the former sections, being employed and married comes with the benefit of greater financial security and thus promotes use of maternal services. Urban residents reported higher rates of skilled birth attendance than women residing in rural areas. This can be owed to urban regions having a greater prevalence of maternal facilities due to greater demand. As seen earlier, higher parity comes with more experience and thus women would be more familiar with the birthing process and could recognize the benefits of receiving a skilled birth attendance. Of intriguing note, single women reported a higher proportion of professional deliveries than their married and insured counterparts. This seemingly exceptional observation was also documented in a similar study [33] which stated that women living independently, and women-head households exercise greater autonomy and decision-making power with regard to using maternal health care services. A comprehensive community-level interventions that consider residential homogeneity regarding infrastructure (e.g., health facilities, roads) and socioeconomic empowerment (e.g., educational and vocational training) could promote women health-promoting behaviours and their access to maternal healthcare services.

Prior studies have revealed that there are significant disparities with respect to the quality of health services between the health sector institutions and between Jordan’s various governorates [16, 17, 32]. This study was no exception. With skilled birth attendance, Mafraq and Jerash scored poorly in comparison to Balga and Amman. The consistent disparities across the governorates is likely due to certain regions being more rural and having a hasher desert climate than others which makes having equipped maternal facilities and trained health workers more challenging. Thus, factors other than health insurance coverage, such as availability, access and quality of maternal services, may pose as the larger underlying issue to MHC services utilization. For example, a qualitative study conducted on Jordanian women regarding their delivery experience reported that maltreatment by health providers impacted their decision-making in choosing to have a midwife over a skilled birth attendant for future pregnancies [34]. Concerted efforts from the regional and national government should be enacted to implement strategies to promote MHC services utilization to bridge the disparity between the advanced governorates and the disadvantaged ones.

### Strengths and limitations

The study used nationally representative data from the DHS, and therefore the findings are generalizable in the study area. However, results of the present study should be discussed in light of its limitations. Firstly, the primary source of the information collected from the study participants was through self-report. This data collection method allows for recall bias which could have resulted in underestimation or overestimation of past experiences, especially given that the chosen variable for parity included a span of 5 years. Second, the study was limited to the variables that were available on the DHS questionnaire; other potentially relevant socio-demographic factors, such as affiliated religion, were not analyzed. Lastly, this study excluded respondents that were not of Jordanian nationality. Given Jordan’s diverse demographic make-up, most notably Syrian and Palestinian refugees that have sought asylum in the country in recent decades [7, 8], it would be of significance to explore this population’s health insurance status and utilization of the essential maternal services. In terms of study design, the study used a cross-sectional research design that limits causal relations between the studied variables. Findings of the study cannot be generalized to all women of reproductive age but only childbearing women. Finally, we acknowledge that there might be some health system factors that affect the variance in the coverage of insurance, and hence MHC services utilization. However, we could not assess this since the dataset does not provide data on it.

## Conclusion

Based on our findings, having health insurance is an enabling resource that promotes maternal healthcare-seeking behaviour. Our study confirms that insured women had increased odds in initiating ANC at the required time, completing of four ANC visits and having a skilled birth attendance. These are all indicators that demonstrate a positive association between health insurance coverage and maternal healthcare services utilization for women in Jordan. This is an important finding in the understanding of improving maternal health in Jordan and globally. Working towards achieving universal health coverage, which is target 3.8 of SGD-3, would impact women positively in seeing greater uptake of essential maternal services. Looking forward, Jordan should consider a cost-effective and equitable plan to account for this population in securing their financial and health status, to especially advance maternal health among its disadvantaged women. Further research should investigate the health insurance and maternal health status on Jordan’s non-citizens, specifically its large Syrian and Palestinian refugee population that are typically excluded from national surveys and the government’s health insurance scheme.

## Data Availability

Data for this study were sourced from Demographic and Health surveys (DHS) and available here: http://dhsprogram.com/data/available-datasets.cfm.

## Abbreviations

ANC: Antenatal Care
HIC: Health Insurance Coverage
MHU: Maternal Health Utilization
SDGs: Sustainable Development Goals
